# What the skull and scapular morphology of the dugong (*Dugong dugon*) can tell us: sex, habitat and body length?

**DOI:** 10.1038/s41598-017-01899-7

**Published:** 2017-05-16

**Authors:** Korakot Nganvongpanit, Kittisak Buddhachat, Patcharaporn Kaewmong, Phaothep Cherdsukjai, Kongkiat Kittiwatanawong

**Affiliations:** 1 Excellence Center in Veterinary Bioscience, Chiang Mai University, Chiang Mai, 50100 Thailand; 2 Animal Bone and Joint Research Laboratory, Department of Veterinary Biosciences and Public Health, Faculty of Veterinary Medicine, Chiang Mai University, Chiang Mai, 50100 Thailand; 3 Department of Biology, Faculty of Science, Naresuan University, Phitsanulok, 65000 Thailand; 4 Phuket Marine Biological Center, Phuket, 83000 Thailand

## Abstract

The dugong (*Dugong dugon*, Müller) is an endangered marine mammal species. We examined the relationship between sex, habitat and body length based on the skull and scapular morphology and morphometrics of 81 dugong samples in Thailand. A total of 58 parameters from the skull and scapula (25 from the cranium, 23 from the mandible and 10 from the scapula) as well as tusks were used in this study. Data were analyzed by univariate analysis, followed by discriminant analysis and multivariate linear regression. Here we show, 100% and 98.5% accuracy rates for sexing using large tusks and the skull, respectively. Scapular morphology using the caudal border tubercle and coracoid process showed 91.30% and 96.15% accuracy rates for identifying males and females. Skull morphometrics could categorize dugong habitat, i.e. living in the Andaman Sea or Gulf of Thailand, with 100% accuracy. Moreover, our model could be used to estimate body length with coefficient of determination (*R* ^2^) of 0.985. The results of our study showed that skull morphology and morphometric measurements could be used as a tool for sex identification, location identification and estimation of body length. But scapular morphology is the best tool for sex identification in dugongs.

## Introduction

The dugong (*Dugong dugon*, Müller) is one of four herbivorous marine mammal species surviving in the family Dugongidae, order Sirenia^1^. Presently, the dugong is an endangered species and has been placed in Appendix I of the Convention on International Trade in Endangered Species (CITES). The highest population of dugongs is found in Australia^2^. In the South to Southeast Asia region, Thailand is the country with the highest population of dugongs^3–6^. Nevertheless, dugongs are rare and are restricted only to some areas along both coastlines of Thailand: the Andaman Sea (i.e. Ranong, Phang Nga, Phuket, Krabi, Trang and Satun) and the Gulf of Thailand (i.e. Rayong, Chanthaburi, Trat and Surat Thani provinces)^4^. The population of dugongs in Thailand was observed in 2000–2001 and reported in 2005. About 200 dugongs were estimated to be living in the Andaman Sea and about 50 in the Gulf of Thailand^3^. The best minimum estimate of population abundance is 123 animals (CV = 60.8%) in Trang province. Due to the limited numbers of this animal, scientists around the world have been trying to study all aspects of dugong biology (i.e. morphology, physiology, ecology and theriology). However, it is very difficult to design a standard study on this animal for many reasons, e.g. the number of animal specimens available at each institute is very low, and because dugongs live in the ocean they cannot be maintained in tanks or aquariums. For these reasons, all studies that are conducted on this animal are very important, even a small study. Moreover, for better understanding of dugong biology, scientists must combine those small studies together.

Animal bone morphology – in particular, skull (cranium plus mandible) morphology – has been studied in many aspects: for example, the diversity of skull morphology within canine species^7^; for species classification in canids and felids^8–10^, and for classifying animals living in different locations, such as captive and wild African lions^11^; for estimating age in African elephants^12^; and for sexing in dogs^13^, cats^14^ and humans^15^. Bones of the dugong have been studied for over 100 years. Based on a literature review by James^16^, skeletal features of the dugong were first described 1902. Subsequent bone morphological studies in dugongs were useful in species identification^17^, sexing^18^, and estimation of age^19, 20^ and body size^21^. However, those studies cannot clearly provide answers for all research questions due to the limitations mentioned above. Thus, more research is still needed to fill gaps in the knowledge about bone morphology.

Some dugong carcasses that are found in the sea or washed up on beaches are decayed, and hence the body size and/or sex is not readily apparent. For this reason, the determination of sex and body length from dugong carcasses is of increasingly greater importance. Because the dugong is an endangered species, this information is used for monitoring the population structure of dugongs in a particular area. Body length can be used to estimate age^22^ and body weight^22, 23^. Moreover, this information, together with autopsy or post-mortem studies, can be useful for investigating or understanding the cause of death, and can be served as a basis for initiating programs to reduce dugong mortality. Although molecular techniques such as polymerase chain reaction (PCR) are available for sex identification^11, 24^, this technique is not widely available in every institute and cannot be used to estimate body length. Consequently, it would be useful if other parameters could be applied as a tool for sex identification and body length estimation. Forty years ago, studies were conducted on skull morphometrics in 52 dugong skulls^25^ and on sexual dimorphism of 32 dugong skulls in North Queensland^18^. The results of the sexual dimorphism study showed that 6 of 26 parameters were significantly different between males and females. However, those parameters did not have clear cut-off values for identifying sex, and the discrimination function from that study was not of high accuracy. From that time until the present there has been no published research on sexual dimorphism of dugong skulls. This is possibly due to limitations on the number of dugong skulls and related records that are available for study. Moreover, Spain *et al*. also noted that the relationship between the development of upper second incisors and skull size possibly could be used to distinguish the sex of mature dugongs^18^. However, they noted that, in the case of female dugongs with fully erupted tusks, this cannot be used for identifying sex.

Therefore, the primary purpose of our study was to use the skull and scapular morphology of dugongs to identify sex, body length and habitat. Information in this study may be beneficial in relation to dugong biology, conservation and forensics. For example, if dugong body remains are found and the sex cannot be identified or the actual body length measured due to decomposition, a method based on the discrimination functions from our study can be used to determine the sex or body length.

## Material and Methods

### Samples and history records

Samples were obtained from the Animal Anatomy Museum, Phuket Marine Biological Center, Phuket, Thailand (Supplement 1). A total of 81 dugong samples were taken from two different habitats, the Andaman Sea (n = 70) and the Gulf of Thailand (n = 11). These samples included skull bones from 37 males, 39 females and 5 of unknown sex. A total of 44 separate permanent tusks were also used in this study, categorized into two sizes, short (length shorter than 7 cm, n = 18) and long tusks (length longer than 7 cm, n = 26). The recorded data used in this study included sex (male or female), body length, and habitat when alive (the Andaman Sea or the Gulf of Thailand).

Animal bones from the Animal Anatomy Museum which were used in this study did not require approval by the Animal Ethics Committee, Faculty of Veterinary Medicine, Chiang Mai University.

### Morphometric measurement parameters and morphology

**Figure 1:**
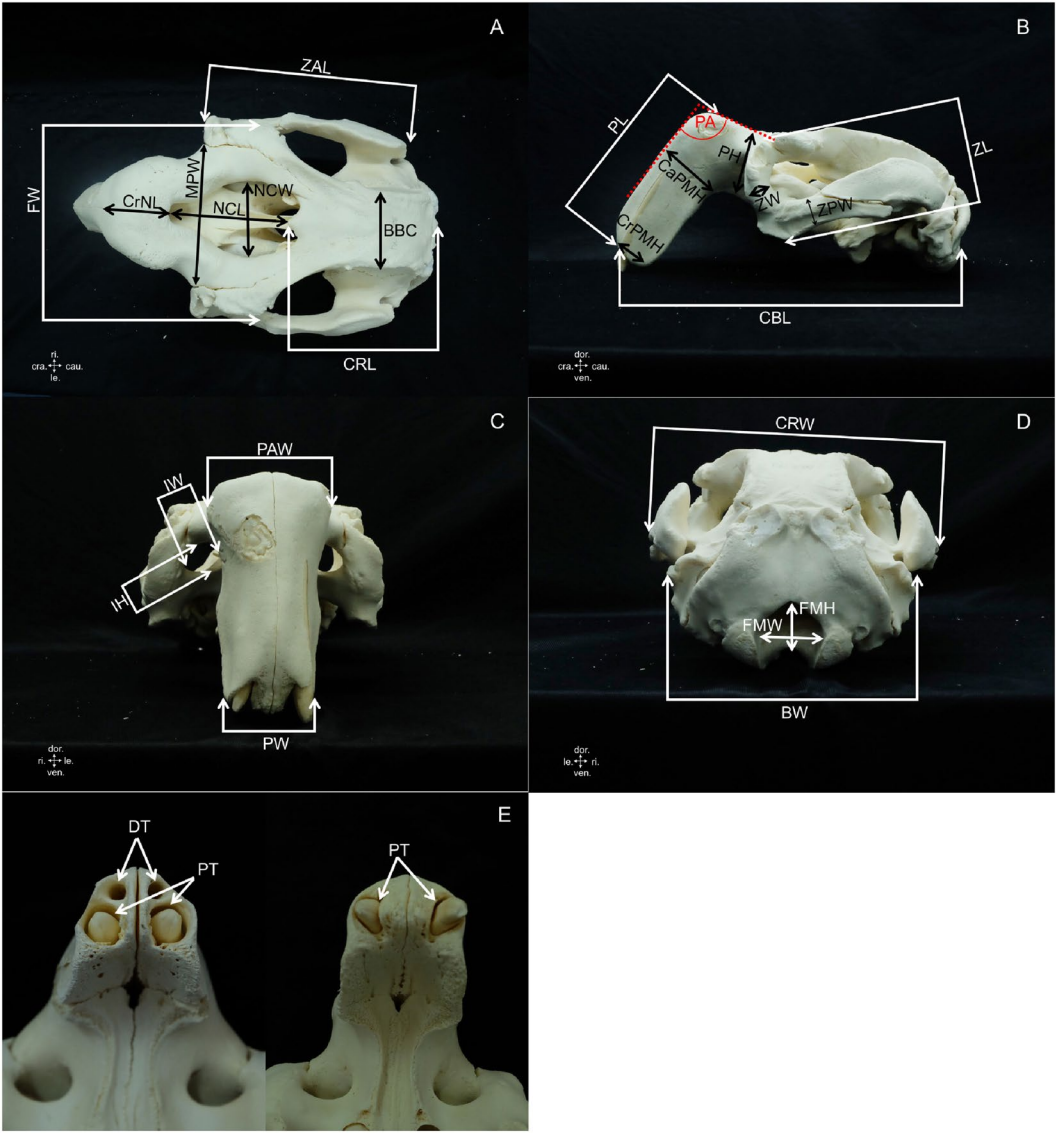
Landmarks for measurements taken from the dugong cranium (see description of measurements in Table 1). Figure 1E, shows a cranium presenting empty sockets of deciduous tusks (DT) and permanent tusks (PT) compared with a cranium presenting only permanent tusks. (cra. = cranial, cau. = caudal, dor. = dorsal, le. = left, ri. = right, ven. = ventral).

**Figure 2:**
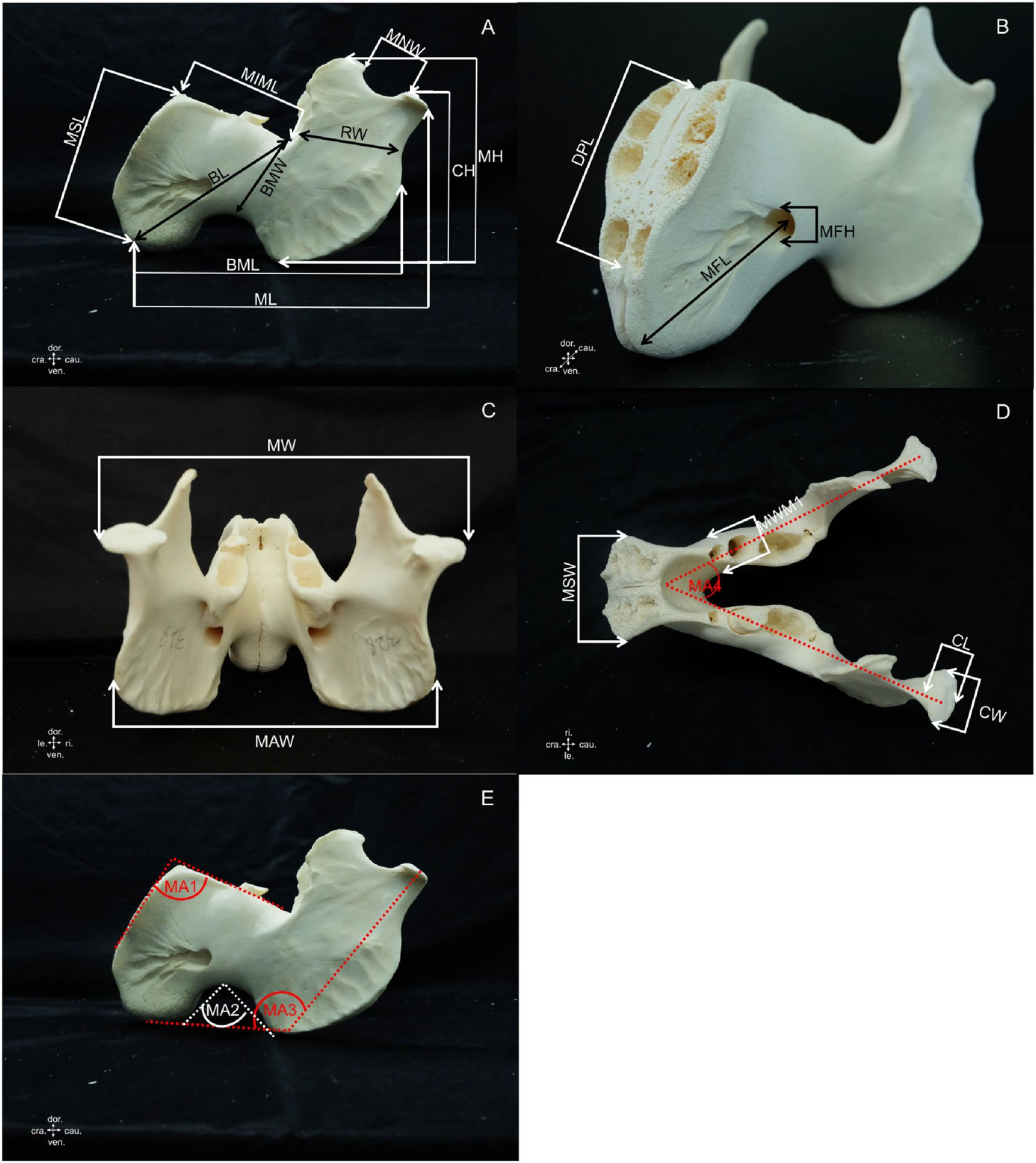
Landmarks for measurements taken from the dugong mandible (see description of measurements in Table 2). (cra. = cranial, cau. = caudal, dor. = dorsal, le. = left, ri. = right, ven. = ventral).

**Figure 3:**
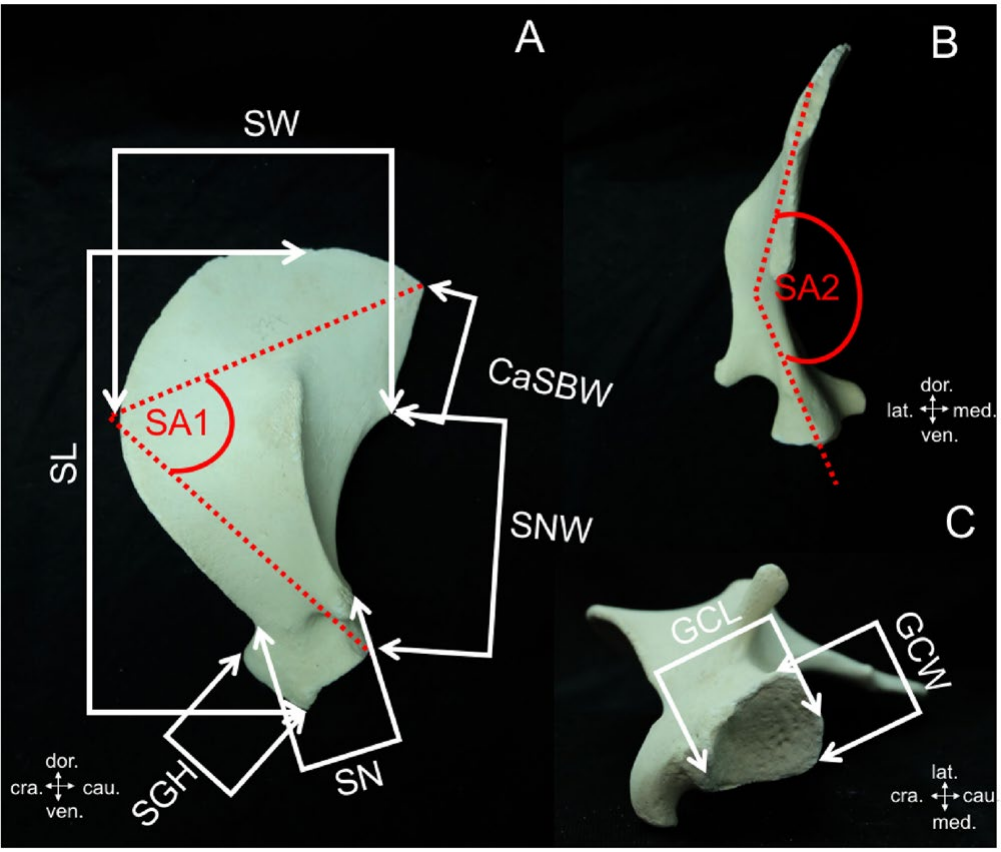
Landmarks for measurements taken from the dugong scapula (see description of measurements in Table 3). (cra. = cranial, cau. = caudal, dor. = dorsal, lat. = lateral, med. = median, ven. = ventral).

A total of 58 parameters were used in this study: 25 parameters from the cranium (Table 1 and Fig. 1), 23 from the mandible (Table 2 and Fig. 2) and 10 from the scapula (Table 3 and Fig. 3). These parameters were adapted from literature reviews of dugongs and other mammal species^8, 10, 11, 18, 26^. Measurements were obtained using either an osteometric board or digital vernier caliper to the nearest 0.1 mm. Each measurement was taken two times, at 48 h intervals, and was recorded as mean value.

**Table 1:** Description of measurements taken from the dugong cranium (see Fig. 1).

Acronym	Measurement	Description of measurements
BBC	Breadth of braincase	Minimum width at the end of the frontal bone across the parietals
BW	Base width	Maximum distance between the right and left mastoid process
CaPMH	Caudal premaxilla height	Height of premaxilla before the point of the angle of premaxilla
CBL	Condylobasal length	Distance from the anterior edge of the premaxilla to the posteriormost projection of the occipital condyle
CRL	Cranial length	Distance from the nuchal crest to the cranial junction of the frontal bone on the median plane
CrPMH	Cranial premaxilla height	Height of premaxilla at the most posterior part of premaxillary bone (after tusk alveolar)
CrNL	Cranial nasal cavity length	Distance from the anterior edge of premaxilla at the angle to the anterior border of the nasal cavity
CRW	Cranial width	Maximum distance between the outer margins of the zygomatic arches
FMH	Foramen magnum height	Maximum foramen magnum height
FMW	Foramen magnum width	Maximum foramen magnum width
FW	Frontal width	Maximum distance between the outer margins of the right and left frontal bones
IH	Infraorbital foramen height	Maximum infraorbital foramen height
IW	Infraorbital foramen width	Maximum infraorbital foramen width
MPW	Maximum premaxilla width	Maximum width of premaxillary bone
NCL	Nasal cavity length	Length of the nasal cavity on the median plane
NCW	Nasal cavity width	Maximum width of the nasal cavity
PA	Premaxilla angle	The angle of premaxillary bone
PAW	Premaxilla angle width	Width at the angle of premaxillary bone
PH	Premaxilla height	Maximum height of premaxillary bone
PL	Premaxilla length	Length of premaxillary bone
PW	Premaxilla width	Width of premaxillary bone at the most posterior part of premaxillary bone after tusk alveolar
ZAL	Zygomatic arch length	Distance from the anterior edge to the posterior edge of the zygomatic arch
ZL	Zygomatic length	Length of the zygomatic bone
ZPW	Zygomatic process width	Minimum width of the zygomatic process of the zygomatic bone
ZW	Zygomatic width	Maximum width of the zygomatic bone

**Table 2:** Description of measurements taken from the dugong mandible (see Fig. 2).

Acronym	Measurement	Description of measurements
BL	Body length	Distance from the anterior edge to the posterior of M3
BML	Body of mandible length	Distance from the anterior edge of the mandible to the posterior end of the mandibular angle
BMW	Body of mandible width	Minimum width of the body of themandible
CH	Condyloid process height	Maximum height from the condyloid process to the lowest border of mandible
CL	Condyle length	Most superior to most inferior portion of condyle
CW	Condyle width	Maximum width from right to left extension of condyle
DPL	Dental pad length	Length of the lower dental pad at the mandibular symphysis
MA1	Mandibular angle 1	Cranial mandibular angle
MA2	Mandibular angle 2	Angle of ventral border of mandible
MA3	Mandibular angle 3	Angle between body and ramus of mandible
MA4	Mandibular angle 4	Angle between left and right mandibles
MAW	Mandibular angle width	Maximum width of the angular process of the mandible measured from the most lateral part of the right to left angular process
MFH	Mental foramen height	Height of the mental foramen
MFL	Mental foramen location	Distance from the anterior edge of the mandible to the mental foramen
MH	Mandible height	Maximum height from the coronoid process to the lowest border of the mandible
MIML	Minimum mandible length	Distance from the cranial mandibular angle to posterior of M3
ML	Mandible length	Distance from the anterior edge of the mandible to the posterior end of jaw condyle
MNW	Mandibular notch width	Maximum width of mandibular notch
MSL	Mandibular symphysis length	Length of the mandibular symphysis
MSW	Mandibular symphysis width	Width of the mandibular symphysis
MW	Mandible width	Maximum width of the mandible measured from right to left condyloid process
MWM1	Mandible width at M1	Width of the mandible at the first molar
RW	Ramus width	Minimum width of the ramus of the mandible

**Table 3:** Description of measurements taken from the dugong scapula (see Fig. 3).

Acronym	Measurement	Description of measurements
CaSBW	Caudal scapular border width	Distance from the caudal angle of the scapula to the superior edge of the caudal scapular notch
GCL	Glenoid cavity length	Maximum length of the glenoid cavity
GCW	Glenoid cavity width	Maximum width of the glenoid cavity
SA1	Scapular angle 1	Angle of the scapula from lateral view
SA2	Scapular angle 2	Angle of the scapula from caudal view
SGH	Supraglenoid tubercle height	Maximum height from highest to lowest border of the supraglenoid tubercle
SL	Scapular length	Maximum length from dorsal border to the lowest ventral angle of the scapula
SN	Scapular narrow	Minimum width of the scapula
SNW	Scapular notch width	Maximum width of the caudal scapular notch
SW	Scapular width	Maximum width of the scapula, measured from the most cranial to caudal scapular border

The morphology of the skull was studied to establish reference hallmarks that could be used to distinguish the skull and scapula of male vs. female dugongs. Moreover, the presence of deciduous tusks was recorded (Fig. 1), and the outer and inner morphology of permanent tusks was observed to compare between males and females as well. For the study of the inner morphology, tusks were cut through the median plane.

### Study design and statistical analysis

The body length of the mature dugong ranges from 2.2–2.4 m. However, some previous studies used dugongs with body lengths of only 2.0 m for their investigation^18, 27^. But our study was not limited to body lengths of 2.0–2.5 m because a good and efficient discrimination function should be able to be applied not only on adults but on immature animals as well. Moreover, some literature has reported that the dugong skull and skeleton continues to grow after puberty^18, 22^. For this reason, in this study we included all skulls and scapulas to create discrimination functions that could be used for sex and habitat identification and body length estimation. All 58 parameters (48 from the skull and 10 from the scapula) were subjected to a Shapiro–Wilk normality test using the R program and were presented as mean ± SD with units in centimeters. Statistical analysis comparing between sexes (male and female) and habitats (Andaman Sea and Gulf of Thailand) was performed using either a *t*-test for normally distributed parameters or Mann–Whitney *U* test for non-normally distributed parameters. All of the parameters in the scapula showed a non-normal distribution, and the data set of mophometrics for dugongs from the Gulf of Thailand, a small population, also resulted in a non-normal distribution. Analysis of differences between habitats was performed using Mann–Whitney *U* test.

To establish equations for sex or habitat determination, we used ratios which represented the proportion of each parameter within the same bone (for example, BCC value was divided by other parameter values of the dugong cranium). In this study, we compared the effectiveness of the morphometrics of the cranium, mandible, skull (cranium and mandible) and scapula in creating a function for assigning sex or habitat. These ratio data sets were analyzed through a stepwise discriminant analysis with leave-one-out classification based on four parts: (i) cranium, (ii) mandible, (iii) skull and (iv) scapula. In order to find correlations between the morphometrics of the skull and scapula and dugong body length, a multivariate linear regression model was used to create an equation for estimating the body length of dugongs based on these parameters of skull and scapular morphology. The average body lengths of dugongs in this study ranged from 0.88 to 3.64 m. In all analyses, a p-value < 0.05 was considered to be statistically significant.

## Results

### Descriptive morphology of the skull, tusk and scapula, comparing males and females

**Figure 4:**
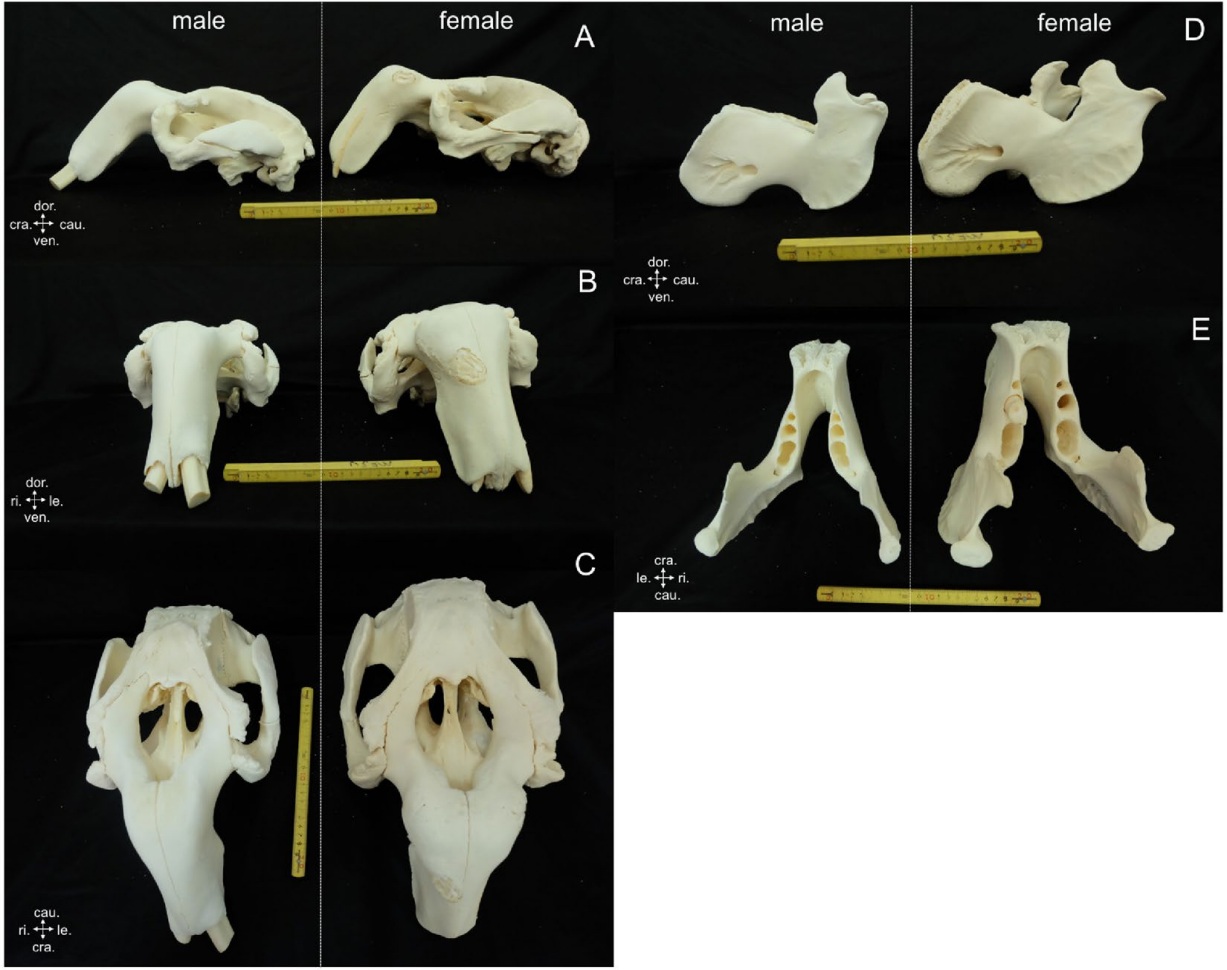
Comparative morphology of the cranium (**A–C**) in male (DU364) and female (DU335) dugongs revealed no significant hallmarks to distinguish males and females: left lateral view (**A**), cranial view (**B**) and dorsal view (**C**). However, the female cranium appears larger than that of the male. Comparative morphology of the male and female mandible, left lateral view (**D**) and dorsal view (**E**), also did not find markers for sex indentification. The female mandible was also larger than the male mandible. (cra. = cranial, cau. = caudal, dor. = dorsal, le. = left, ri. = right, ven. = ventral).

The skull (cranium and mandible) morphology of males and females was similar, and no significant hallmarks were found that could be used as sex characteristics. However, male skulls were smaller than female skulls, even though the body was larger or longer. An example is shown in Fig. 4, comparing the cranium and mandible of a male dugong whose body length was 3.64 m with a female dugong with body length of 2.59 m.

The existence of the deciduous tusks for dugongs was explored. Overall, 41% of males and 54% of females presented deciduous tusks. However, 100% of dugongs with body size more than 2.31 m did not presented deciduous tusks while 77% of dugongs with body length less than 2.31 m presented deciduous tusks.

**Figure 5:**
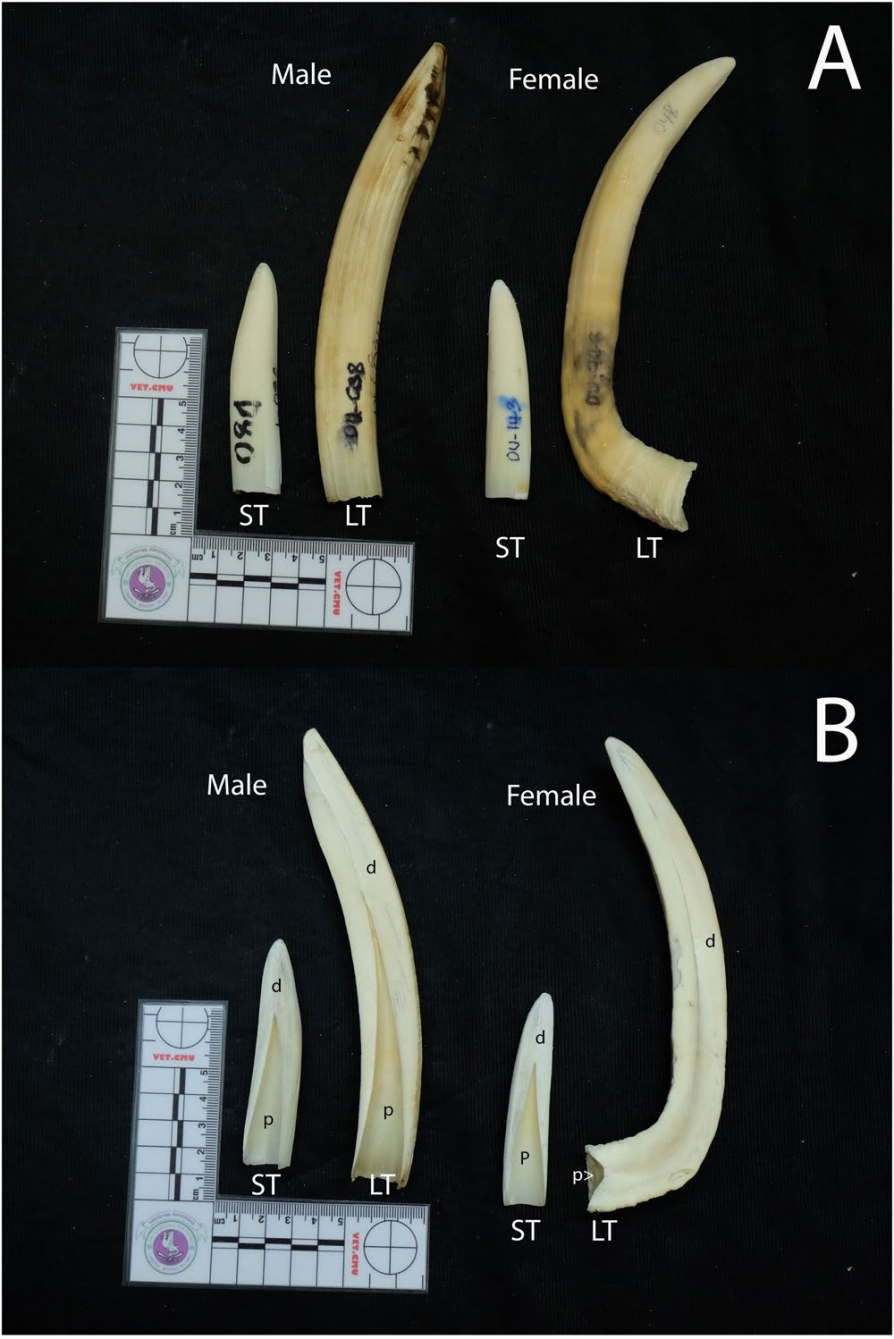
Comparative morphology of small tusks (ST) and large tusks (LT) in male and female dugongs, left lateral view (**A**) and median plane section (**B**). It was difficult to use small tusks for sex identification, even when observing median plane sections; dental pulp (p) was similar between both sexes. In large permanent tusks there was a clear difference between sexes; dental pulp in males was triangle-shaped (similar to small tusks), but female tusks were filled with dentine, with only a small amount of dental pulp.

Both outside and median plane sections of small tusks in males and females were similar (Fig. 5). The median plane showed dental pulp in a triangular shape in both sexes. The proximal part of large tusks in females was more convex than in males, and the median plane view showed a clear difference between sexes. Male tusks had dental pulp in a triangular shape, while females had a small amount of dental pulp. From this morphology, large permanent tusks of dugongs showed clear differences between males and females, with 100% accuracy from a total of 27 large permanent tusks. While small permanent tusks were not able to be used for sex identification, 39% (7/18) were accurately identified as male tusks, while none could be identified as tusks from females.

**Figure 6:**
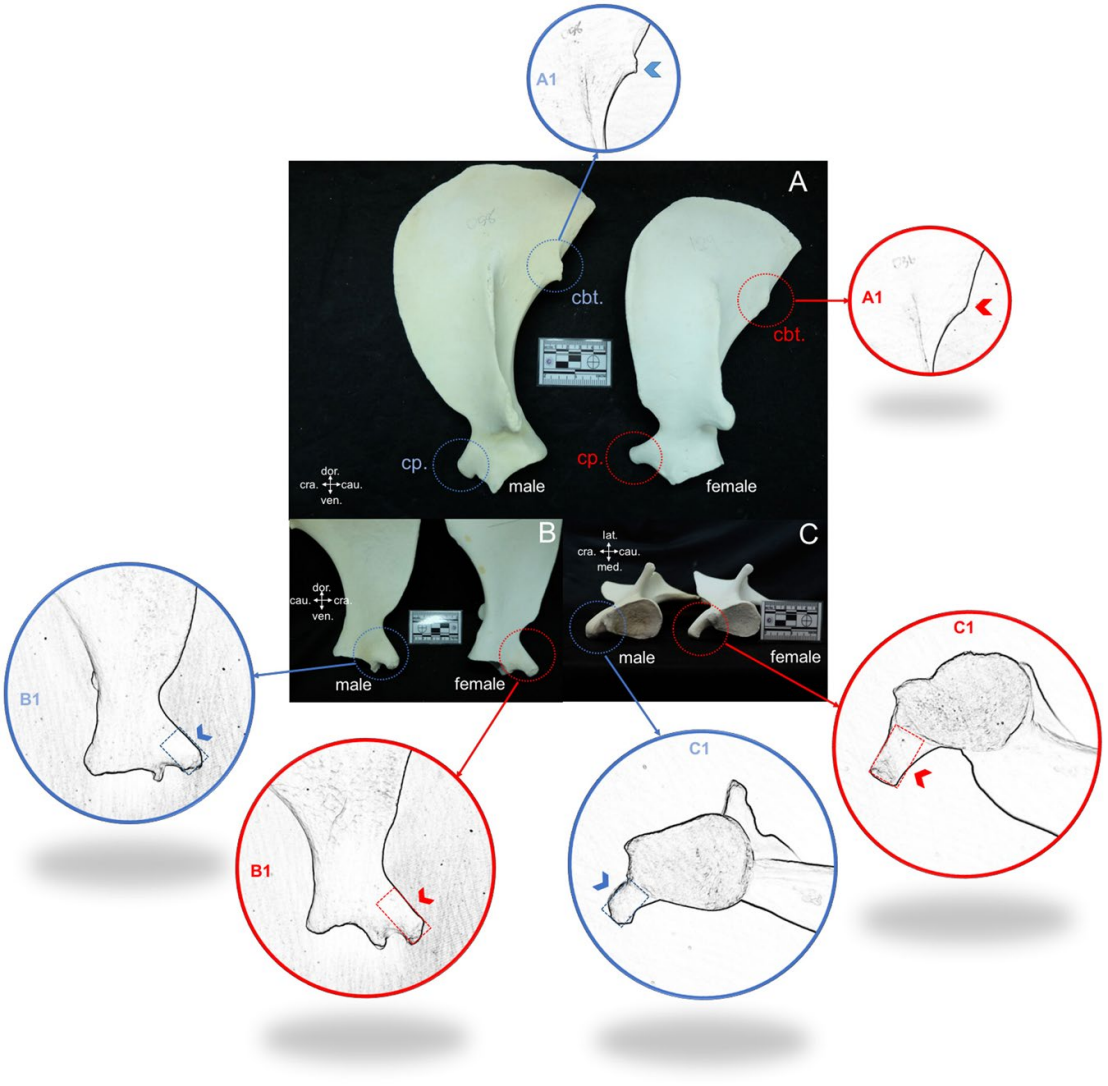
Lateral view (**A**), medial view (**B**) and ventral view (**C**) comparing scapulas of male (DU058) and female (DU129) dugongs revealed two significant hallmarks that could be used for sex identification: caudal border tuberosity (cbt.) and coracoid process (cp.) (**A**). In males, the scapula presented a caudal border tuberosity (A1), while in females this was not present (A2). The shape of the coracoid process in females is smaller and longer (B1,C1), while in males it is shorter and thicker (B2,C2). (cra. = cranial, cau. = caudal, dor. = dorsal, lat. = lateral, med. = median, ven. = ventral).

The scapula of the dugong is flat with a triangular shape, slightly curved inward toward the medial surface. This study found two significant hallmarks on the scapula that could be used as a tool for sex identification in dugongs (Fig. 6). The first was on the caudal boarder of the scapula: males presented a caudal border tuberosity, which was absent in females. The second was on the coracoid process: in males this presented as a short and thick shape, while in females it presented as longer and thinner compared with males. These two characteristics had a 91.30% (21/23) accuracy rate for identifying males and a 96.15% (25/26) accuracy rate for females. However, we note that these two characteristics can be used only in mature scapulas (all epiphyses on the scapula are closed).

### Morphometric correlation

**Figure 7:**
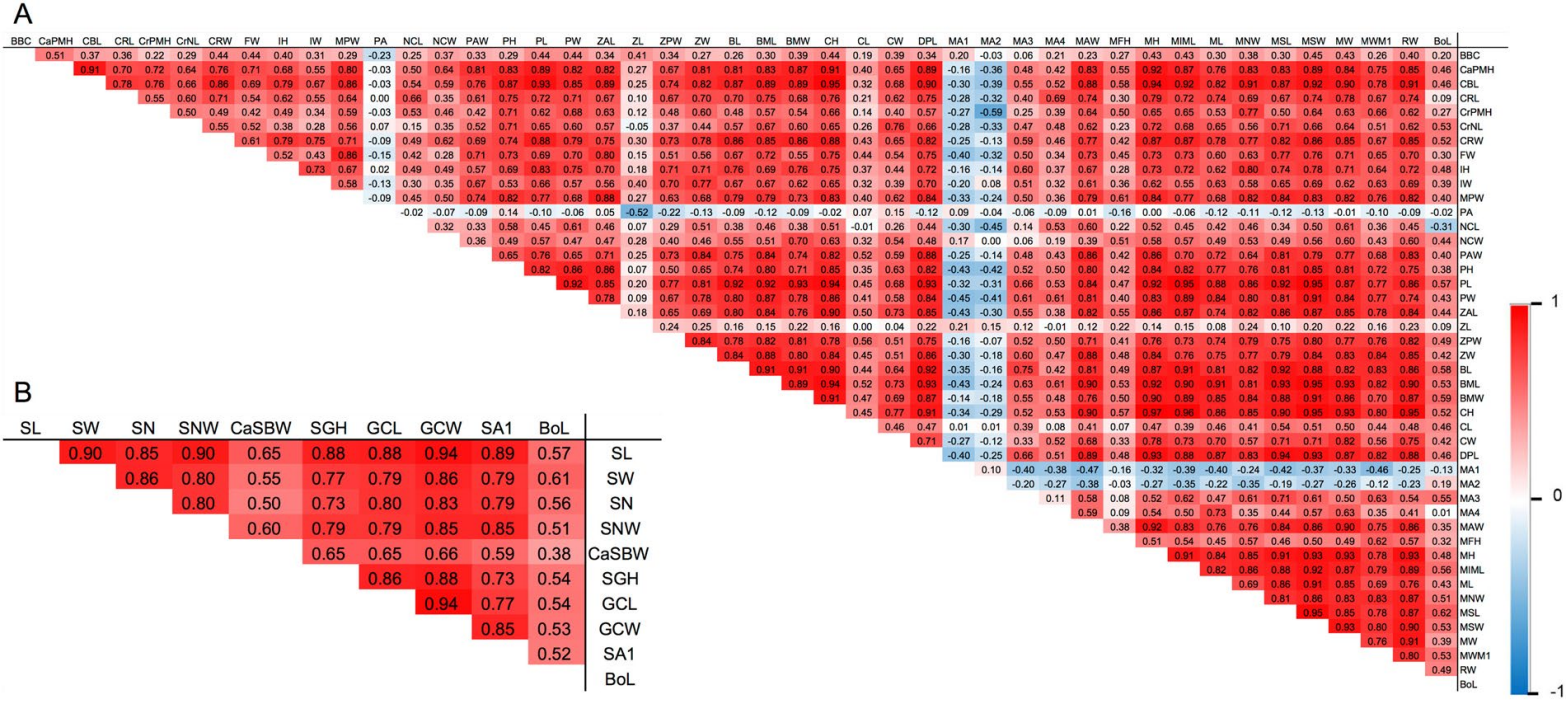
Pearson’s correlation coefficients between each parameter of dugong skulls (**A**) and scapulas (**B**). BoL represents body length.

**Figure 8:**
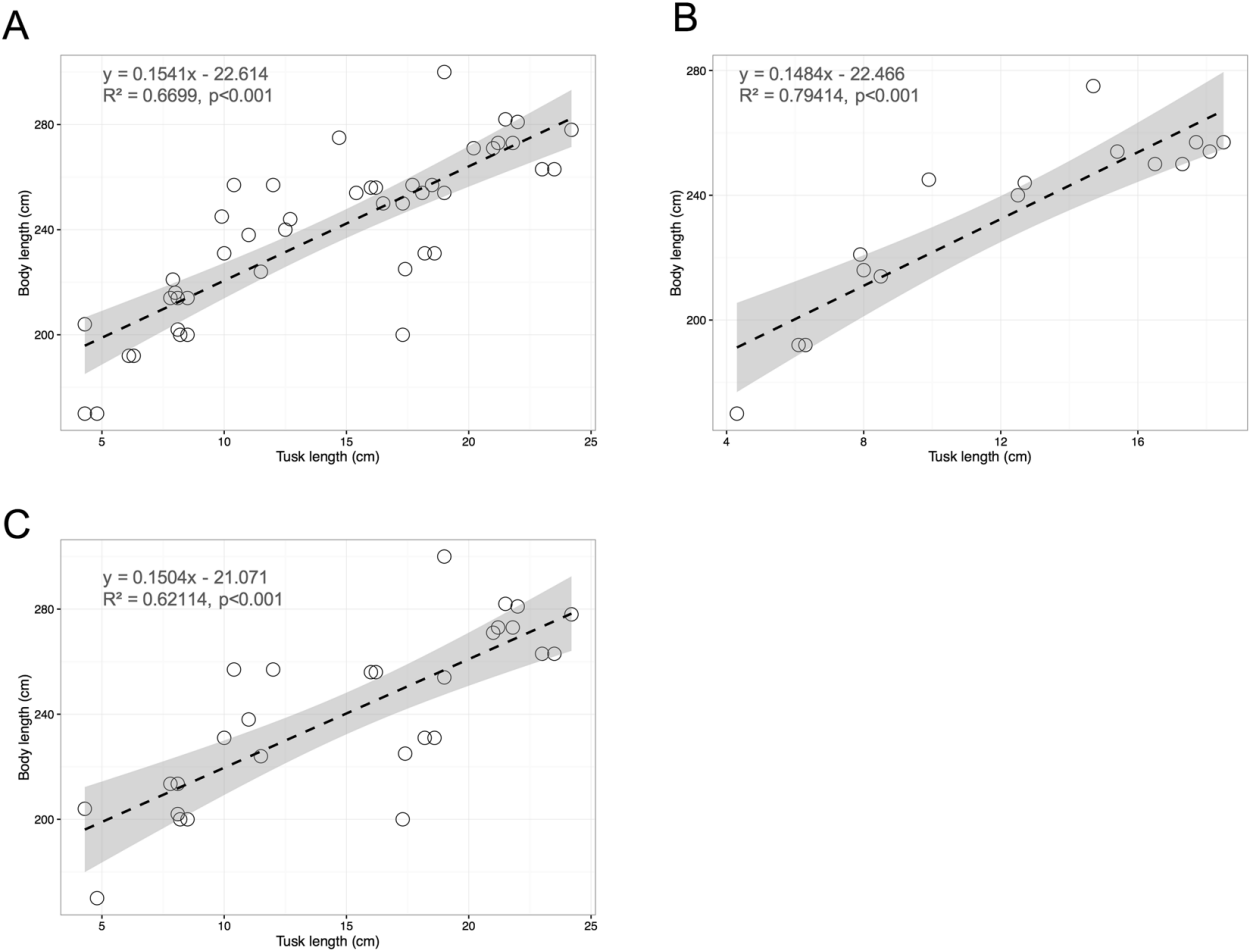
The relationship of dugong tusk length (cm) and body length (cm) for both sexes (**A**), only males (**B**) and only females (**C**). The dashed line is the regression line of each equation with 95% confidence interval. Tusk length was compared between sexes, and no significant difference was found (p = 0.1024).

The correlation among the parameters of skull bones, including cranium and mandible, was performed by Pearson’s correlation analysis (Fig. 7A). It was found that most correlations of these parameters were positive, with the highest positive relationship observed between MH and CH. A negative relationship could be found between MA1, MA2 and PA and the others; the highest negative relationship was noted between MA2 and CrPMH. Some parameters of skull morphometrics had a linear relationship with the body length of dugongs, especially MSL and BMW with 0.62 and 0.59, respectively. All parameters among scapular morphometrics showed a positive relationship; GCW and SL showed the highest relationship with 0.94 (Fig. 7B). The highest correlation of scapular morphometrics and body length was 0.61, for SW. However, SA2 was excluded from this correlation analysis because some values were missing. Moreover, we could not find a relationship (p = 0.1024) between dugong tusk length and body length for either sex (Fig. 8).

### Sex determination using skull and scapular morphometric equations

**Figure 9:**
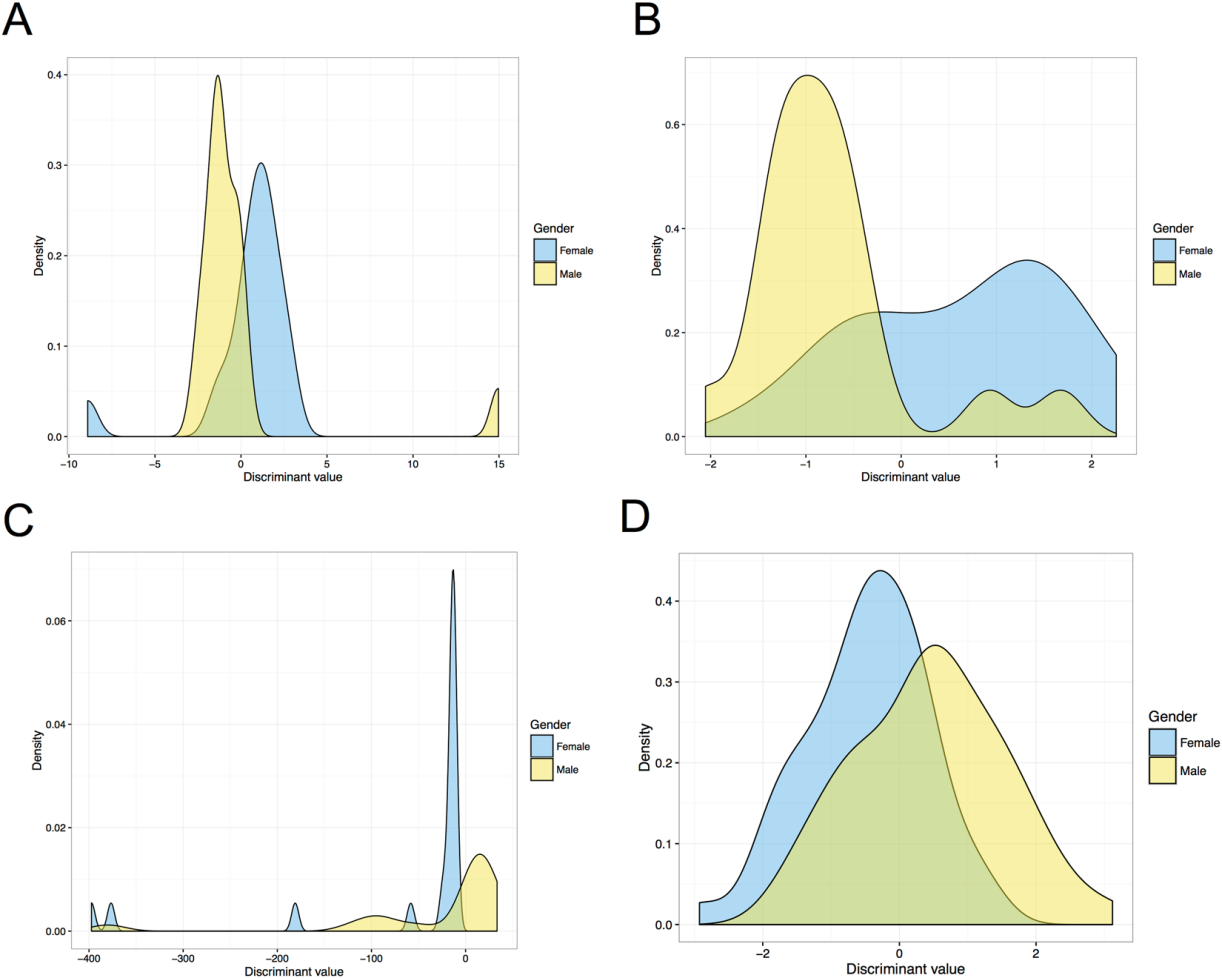
The distribution of discriminant values between males and females, based on the morphometrics of the cranium (**A**), mandible (**B**), skull (**C**) and scapula (**D**).

A total of 76 dugong samples, including males (n = 37) with body length between 1.16–3.64 m (2.17 ± 0.46) and females (n = 39) with body length between 0.88–2.81 m (2.26 ± 0.46), were included in this study. Another 4 dugongs were excluded due to unknown sex. The morphometric measurements proved that both the cranium and mandible from males were smaller compared with females. Out of a total of 48 parameters (Table 4) used to compare between males and females, 29 of 43 length parameters for females were higher (p > 0.05) than for males, and 4 of 5 angles for males were higher than for females. However, there was no significant difference of any parameters in both the cranium and mandible, while scapular morphometrics had two significant variables, SW and SNW, which showed a remarkable difference between males and females (Table 3). Ratios obtained from morphometrics of the cranium, mandible and skull were analyzed by stepwise discrimination to generate equations for sex determination; each equation consisted of three, two and thirteen variables for the cranium, mandible and skull, respectively (Table 5). As shown in Fig. 9, the distribution of discriminant values (DV) obtained from the equations and the boundary decision for sexing was set at zero of DV. The DV distribution of the cranium, mandible and scapula had large overlapping areas between males and females (Fig. 9A,B,D), whereas that of the skull had a smaller overlapping area (Fig. 9C). The skull equation had the highest accuracy (96.7%) and precision (96.9%); all males were correctly determined, while females had an incorrect prediction. In contrast, the equation obtained from the scapula displayed the lowest accuracy (68.1%) and precision (68.1%) (Table 5).

**Table 4:** Measurement data (mean ± SD) taken from dugong skulls (cranium and mandible) and scapulas, by sex.

Cranium				Mandible				Scapula			
Acronym	Male	Female	p-value	Acronym	Male	Female	p-value	Acronym	Male	Female	p-value
BBC	6.30 ± 0.82	6.55 ± 0.59	0.28	BL	14.78 ± 1.59	14.69 ± 2.13	0.88	SL^*^	21.82 ± 4.19	23.58 ± 3.14	0.07
BW	16.28 ± 1.00	16.33 ± 0.95	0.91	BML^*^	20.73 ± 2.09	20.81 ± 2.71	0.91	SW^*^	10.94 ± 1.94	11.97 ± 1.84	**0.02**
CaPMH^*^	6.91 ± 1.75	6.86 ± 0.77	0.42	BMW	6.42 ± 0.73	6.34 ± 1.01	0.78	SN^*^	3.60 ± 0.61	3.91 ± 0.60	0.06
CBL	32.42 ± 2.75	32.89 ± 3.88	0.68	CH	13.94 ± 1.47	14.01 ± 2.00	0.91	SNW^*^	10.34 ± 1.70	11.29 ± 1.20	**0.01**
CRL	15.40 ± 1.55	16.03 ± 2.41	0.33	CL	1.85 ± 0.21	1.81 ± 0.32	0.67	CaSBW^*^	6.55 ± 3.17	6.73 ± 1.44	0.27
CrPMH	4.78 ± 0.77	4.61 ± 0.66	0.46	CW^*^	1.83 ± 0.30	1.97 ± 0.42	0.52	SGH^*^	2.93 ± 1.06	3.04 ± 0.79	0.77
CrNL^*^	1.77 ± 0.57	1.86 ± 0.51	0.55	DPL	8.23 ± 1.09	8.38 ± 1.53	0.72	GCL^*^	4.03 ± 1.11	4.02 ± 0.67	0.58
CRW	19.12 ± 2.11	19.22 ± 2.33	0.88	MA1^#,*^	113.47 ± 5.04	113.38 ± 4.41	0.73	GCW^*^	3.29 ± 0.78	3.43 ± 0.53	0.50
FMH	4.06 ± 0.46	4.03 ± 0.42	0.89	MA2^#,*^	113.63 ± 6.76	111.90 ± 6.18	0.46	SA1^*^	65.31 ± 8.83	68.52 ± 7.23	0.06
FMW	4.00 ± 0.24	4.13 ± 0.22	0.25	MA3^#,*^	123.37 ± 4.57	123.19 ± 5.47	0.62	SA2^*^	156.25 ± 10.69	150.00 ± 11.87	0.21
FW	14.43 ± 1.67	14.98 ± 1.89	0.33	MA4^#,*^	52.37 ± 9.19	56.38 ± 6.71	0.18				
IH	2.47 ± 0.31	2.53 ± 0.27	0.57	MAW	15.87 ± 1.50	16.17 ± 2.24	0.63				
IW	3.14 ± 0.39	3.24 ± 0.48	0.47	MFH	1.35 ± 0.26	1.31 ± 0.25	0.60				
MPW	11.58 ± 1.43	12.17 ± 1.69	0.25	MFL	8.06 ± 0.83	8.12 ± 1.09	0.86				
NCL^*^	9.58 ± 1.04	10.41 ± 2.99	0.36	MH	14.65 ± 1.40	14.97 ± 2.09	0.58				
NCW	6.77 ± 0.61	6.72 ± 0.71	0.80	MIML^*^	9.22 ± 1.04	8.97 ± 1.17	0.41				
PA^#,*^	111.45 ± 4.85	109.88 ± 3.66	0.78	ML	23.69 ± 2.54	23.57 ± 3.03	0.90				
PAW	7.17 ± 0.70	7.84 ± 1.23	0.06	MNW	5.26 ± 0.72	5.19 ± 0.87	0.78				
PH	7.23 ± 1.17	7.33 ± 1.18	0.78	MSL	10.54 ± 1.33	10.73 ± 2.06	0.74				
PL^*^	15.30 ± 1.5	15.53 ± 1.95	0.79	MSW	5.59 ± 1.00	5.49 ± 1.14	0.76				
PW	4.93 ± 1.02	4.94 ± 1.10	0.97	MW	15.92 ± 1.65	15.84 ± 1.82	0.89				
ZAL	16.07 ± 1.56	16.51 ± 2.06	0.47	MWM1^*^	1.96 ± 0.52	1.90 ± 0.35	0.76				
ZL^*^	8.36 ± 1.68	8.18 ± 0.85	0.67	RW	6.70 ± 0.68	6.71 ± 0.95	0.96				
ZPW	2.94 ± 0.28	2.98 ± 0.41	0.70								
ZW	2.11 ± 0.24	2.29 ± 0.42	0.11								

All parameters are in cm, except superscript ^#^indicates units in degrees. ^*^Non-normally distributed parameter using Mann-Whitney *U* test for testing a significant difference.

**Table 5:** Functions obtained from stepwise discriminant analysis based on the morphometrics of the cranium, mandible, skull and scapula and their classification results for assigning gender.

Bone	Variable	Function coefficients	Sex	Percentage of prediction^a^		Accuracy	Precision
				Male	Female		
Cranium	CRL/CBL	46.063	Male	87.5	12.5	81.8	82.2
	CaPMH/CRL	25.906	Female	23.5	76.5		
	PAW/PL	22.616					
	(Constant)	−43.972					
Mandible	CW/CL	3.607	Male	88.9	11.1	78.4	79.7
	MH/MIML	12.118	Female	31.6	68.4		
	(Constant)	−23.604					
Skull	FW/BBC	3.345	Male	100	0	96.7	96.9
	ZL/BBC	17.915	Female	6.7	93.3		
	CRL/CBL	−228.400					
	CBL/IW	76.844					
	NCW/PA	463.990					
	CRW/PAW	16.807					
	IW/PAW	−42.485					
	ZW/ZAL	1292.776					
	ZAL/ZW	27.766					
	MA1/MA2	−47.992					
	ML/MFH	1.002					
	MSW/MIML	−39.484					
	DPL/MNW	−58.880					
	(Constant)	−430.757					
Scapula	GCL/SW	18.205	Male	70.21	29.79	68.1	68.1
	SA1/SNW	1.580	Female	34.10	65.90		
	(Constant)	−16.236					

^a^Cross-validation is done only for those cases in the analysis. In cross-validation, each case is classified by the functions derived from all cases other than that case.

### Habitat identification using skull and scapular morphometric equations

A total of 80 dugongs were taken from two different habitats – 69 dugongs from the Andaman Sea (male = 33, female = 33, sex unknown = 4, and body length = 2.20 ± 0.48 m) and 11 dugongs from the Gulf of Thailand (male = 4, female = 6, sex unknown = 1, and body length = 2.21 ± 0.28 m). One dugong was excluded from the study due to missing habitat records.

**Figure 10:**
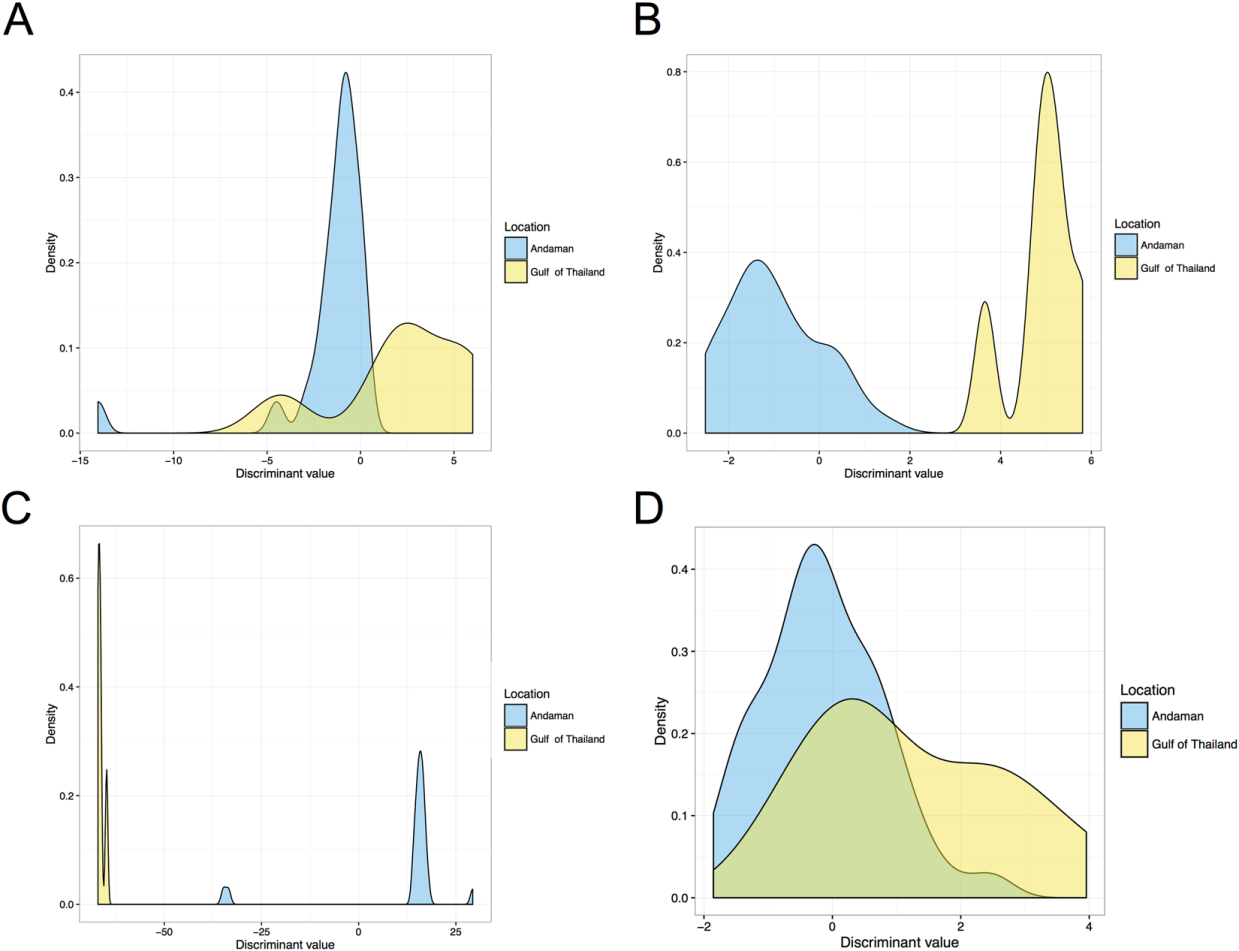
The density of discriminant values between the Andaman Sea and the Gulf of Thailand, based on morphometrics of the cranium (**A**), mandible (**B**), skull (**C**) and scapula (**D**).

Out of a total of five parameters, three parameters from the cranium (BW, NCW, ZAL) and one parameter from the mandible (CW) had significantly higher values in skulls from the Andaman Sea, while one parameter from the mandible (CL) showed a significantly higher value in skulls from the Gulf of Thailand (Table 6). In the scapula, we found that SN exhibited a significant difference, i.e. greater in dugongs from the Andaman Sea. As shown in Table 7, three equations from a stepwise discriminant analysis based on the morphometrics of the cranium, mandible and skull, included five, six and sixteen variables, respectively. The boundary decision for habitat determination was zero of DV. The distributions of DV from three equations are displayed in Fig. 10; overlapping areas were observed for the cranium and scapula but not for the mandible and skull. The results of habitat identification based on morphology revealed that the skull equation possessed the highest accuracy (100%) and precision (100%), while the cranium and mandible equations had an accuracy of 93.8% and 97.3%, respectively. The scapula appeared to have a low accuracy for identifying habitat.

**Table 6:** Measurement data (mean ± SD) taken from dugong skulls (cranium and mandible) and scapulas from two habitats (the Andaman Sea and the Gulf of Thailand).

Cranium				Mandible				Scapula			
Acronym	Andaman	Gulf of Thai	p-value	Acronym	Andaman	Gulf of Thai	p-value	Acronym	Andaman	Gulf of Thai	p-value
BBC	6.50 ± 0.90	6.11 ± 0.93	0.29	BL	14.75 ± 1.89	14.20 ± 2.05	0.52	SL	22.8 ± 3.95	22.32 ± 2.45	0.29
BW	16.66 ± 0.90	15.32 ± 0.54	**0.02**	BML	20.81 ± 2.45	20.12 ± 2.34	0.52	SW	11.54 ± 1.95	10.69 ± 1.64	0.21
CaPMH	6.96 ± 1.39	6.51 ± 1.09	0.32	BMW	6.40 ± 0.89	6.10 ± 0.86	0.24	SN	3.81 ± 0.65	3.40 ± 0.46	**0.05**
CBL	32.79 ± 3.34	31.25 ± 3.34	0.3	CH	14.03 ± 1.78	13.38 ± 1.55	0.27	SNW	10.82 ± 1.53	10.77 ± 1.42	0.82
CRL	15.81 ± 1.96	14.95 ± 1.89	0.3	CL	1.81 ± 0.28	2.01 ± 0.11	**0.04**	CaSBW	6.57 ± 1.75	7.77 ± 5.82	0.57
CrPMH	4.73 ± 0.73	4.45 ± 0.66	0.51	CW	1.94 ± 0.37	1.65 ± 0.21	**0.03**	SGH	2.97 ± 0.95	3.19 ± 0.41	0.88
CrNL	1.88 ± 0.55	1.44 ± 0.23	0.05	DPL	8.29 ± 1.34	8.20 ± 1.34	0.69	GCL	4.03 ± 1.00	4.00 ± 0.58	0.51
CRW	19.37 ± 2.33	18.26 ± 1.46	0.22	MA1^#^	113.49 ± 4.77	114.17 ± 4.75	0.56	GCW	3.40 ± 0.71	3.21 ± 0.42	0.26
FMH	4.10 ± 0.45	3.99 ± 0.45	0.85	MA2^#^	112.49 ± 6.68	115.33 ± 4.68	0.24	SA1	67.31 ± 8.22	63.9 ± 7.34	0.16
FMW	4.04 ± 0.22	4.17 ± 0.27	0.32	MA3^#^	123.11 ± 5.10	122.33 ± 6.50	0.87	SA2	151.39 ± 11.14	162.50 ± 8.66	0.07
FW	14.91 ± 1.84	13.74± 1.52	0.1	MA4^#^	54.43 ± 8.54	54.00 ± 4.90	0.78				
IH	2.50 ± 0.30	2.40 ± 0.25	0.44	MAW	15.96 ± 1.94	15.86 ± 2.14	0.82				
IW	3.17 ± 0.45	3.29 ± 0.34	0.51	MFH	1.36 ± 0.25	1.19 ± 0.20	0.14				
MPW	12.02 ± 1.58	10.97 ± 1.15	0.09	MFL	8.13 ± 0.95	7.69 ± 1.09	0.23				
NCL	10.02 ± 2.35	9.66 ± 1.16	0.8	MH	14.87 ± 1.79	14.25 ± 1.78	0.42				
NCW	6.84 ± 0.64	6.32 ± 0.36	**0.04**	MIML	9.17 ± 1.12	8.36 ± 0.96	0.07				
PA^#^	110.82 ± 4.61	110.83 ± 2.04	0.87	ML	23.65 ± 2.84	23.38 ± 2.29	0.97				
PAW	7.44 ± 0.98	7.78 ± 1.32	0.56	MNW	5.27 ± 0.82	4.77 ± 0.53	0.13				
PH	7.43 ± 1.16	6.50 ± 0.85	0.06	MSL	10.66 ± 1.75	10.14 ± 1.81	0.42				
PL	15.37 ± 1.75	15.19 ± 1.86	0.88	MSW	5.56 ± 1.08	5.22 ± 0.96	0.4				
PW	4.94 ± 1.07	4.86 ± 0.92	0.95	MW	15.93 ± 1.79	15.32 ± 1.16	0.45				
ZAL	16.54 ± 1.81	14.89 ± 1.63	**0.04**	MWM1	1.94 ± 0.46	1.76 ± 0.23	0.28				
ZL	8.31 ± 1.38	8.20 ± 0.91	0.78	RW	6.75 ± 0.85	6.33 ± 0.54	0.23				
ZPW	2.97 ± 0.34	2.89 ± 0.36	0.54								
ZW	2.19 ± 0.33	2.20 ± 0.45	0.72								

All parameters are in cm, except superscript ^#^indicates units in degrees.

**Table 7:** Functions obtained from stepwise discriminant analysis based on the morphometrics of the cranium, mandible, skull and scapula, and classification results for predicting habitat.

Bone	Variable	Function coefficients	Habitats	Percentage of prediction^a^		Accuracy	Precision
				Andaman Sea	Gulf of Thailand		
Cranium	PW/BBC	10.616	Andaman Sea	96.2	3.8	93.8	89.7
	PA/CBL	2.848	Gulf of Thailand	16.7	83.3		
	PA/CrNL	0.045					
	IW/PH	9.425					
	PAW/ZAL	21.732					
	(Constant)	−35.429					
Mandible	CL/CW	5.111	Andaman Sea	96.8	3.2	97.3	92.8
	CL/MAW	−1076.756	Gulf of Thailand	0	100		
	MIML/MAW	273.618					
	MSW/MAW	−155.062					
	CL/MIML	940.45					
	CL/MSW	−187.425					
	(Constant)	−111.092					
Skull	CrNL/CRL	163.055	Andaman Sea	100	0	100	100
	PA/CRL	−15.284	Gulf of Thailand	0	100		
	PW/CRW	−105.309					
	IH/IW	173.012					
	IW/NCW	114.274					
	NCW/PW	50.157					
	CaPMH/ZPW	36.154					
	PL/ZW	−8.489					
	MWM1/BML	−4772.791					
	CL/CW	−61.507					
	CH/MA1	732.804					
	MNW/MAW	1075.451					
	MA1/MH	19.541					
	BMW/MIML	−473.96					
	MWM1/MNW	463.435					
	ML/MWM1	−9.99					
	(Constant)	22.908					
Scapula	SNW/SN	2.352	Andaman Sea	76.40	23.60	73.7	61.5
	SGH/SN	3.427	Gulf of Thailand	50	50		
	(Constant)	−9.510					

^a^Cross-validation is done only for those cases in the analysis. In cross-validation, each case is classified by the functions derived from all cases other than that case.

### Body length determination using skull morphometric equation

**Figure 11:**
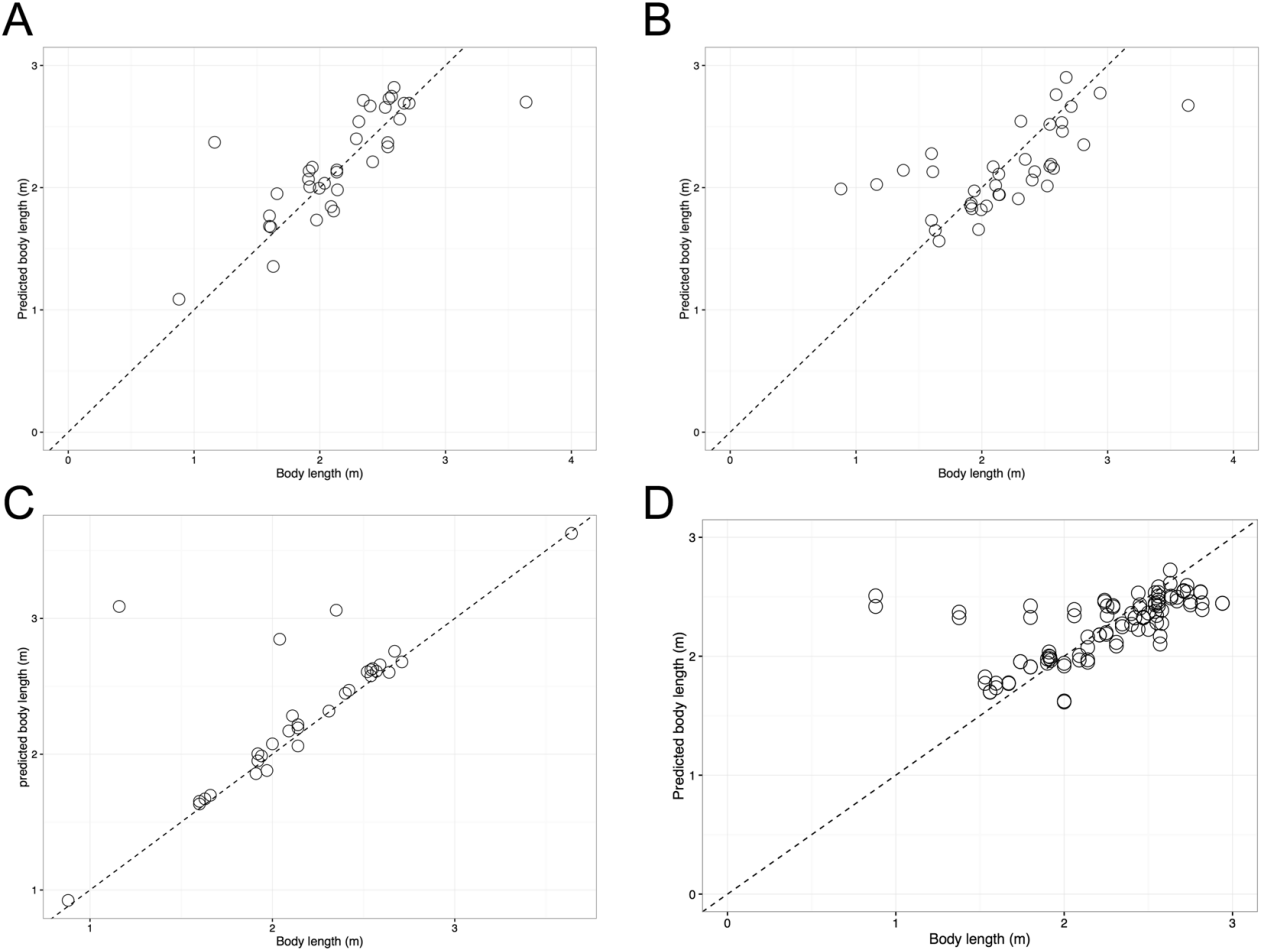
Relationship between body length of dugongs (in meters) and the predicted body length through an effective function for estimating body length of dugongs based on the morphometrics of the cranium (**A**), mandible (**B**), skull (**C**) and scapula (**D**). The dashed line represents the diagonal line where *x* is equal to *y*.

The average body length of 80 dugongs was 2.20 ± 0.46 m, ranging from 0.88 to 3.64 m. One dugong was excluded from the study because the record of body length was missing. According to the correlations between body length and other parameters (Fig. 7), most did not exceed a moderate correlation. To acquire an effective equation for estimation of dugong body length, multivariate linear regression was performed based on the morphometrics of the cranium, mandible and scapula. As shown in Table 8, the fittest model with the highest adjusted correlation coefficient of 0.985 was a skull-based equation, followed by the cranium, mandible and scapula with correlation coefficients of 0.746, 0.381 and 0.371, respectively. The error of the skull-based equation was 6.5%. In the skull-based equation, all 12 variables displayed a significant coefficient at p < 0.05. Figure 11 illustrates the agreement between body length and predicted body length obtained from each equation. We found that almost all of the values in the skull-based equation were on the diagonal line, indicating high accuracy and precision.

**Table 8:** The coefficients and coefficients of correlation of linear regression for estimating the body length of dugongs, based on the cranium, mandible, skull and scapula.

Bone	Variable	Unstandardized coefficients			R^2^	Adjusted R^2^	Standard error of the estimate
		Beta	Std. error	p-value			
Cranium	PL	0.286	0.033	0	0.765	0.746	0.2685
	NCL	−0.144	0.024	0			
	(Constant)	−0.794	0.431	0.078			
Mandible	MSW	0.228	0.085	0.011	0.434	0.381	0.4131
	CL	0.616	0.294	0.044			
	MA2	0.025	0.012	0.051			
	(Constant)	−3.053	1.568	0.06			
Skull	MSL	0.300	0.029	0	0.992	0.985	0.0655
	NCL	−0.181	0.009	0			
	PW	0.463	0.035	0			
	NCW	0.173	0.033	0			
	ML	−0.109	0.014	0			
	FW	−0.049	0.013	0.002			
	DPL	−0.341	0.041	0			
	MAW	0.086	0.021	0.001			
	MA2	0.020	0.003	0			
	CrPMH	0.131	0.034	0.002			
	CRW	0.049	0.014	0.004			
	CL	−0.154	0.063	0.029			
	(Constant)	−1.280	0.458	0.015			
Scapula	SW	0.137	0.018	0	0.378	0.371	0.342
	(Constant)	0.675	0.209	0.002

## Discussion

The results from this study have implications for dugong conservation, ecology and osteology, and can be used to monitor changes in the population structure, i.e. the number, size and sex of dugongs. Sex and body length frequency distribution can provide life-table information on living dugongs. Moreover, the results of this study expanded the basic knowledge of dugong osteology, revealing sexual dimorphism in the skull and scapula. For example, estimation of the sex ratio of the dugong population would provide the current status of the dugong, leading to the creation of an appropriate conservation plan. Since the first published report on the morphometric differences between male and female dugongs in 1976, no studies have been performed on this topic. The highlight of our study was demonstrating that skull morphometrics could distinguish between males and females with a 96.9% accuracy rate, while using large permanent tusks gave 100% accuracy. Skull measurements were different between dugongs living in the Andaman Sea and the Gulf of Thailand with 100% accuracy. Moreover, some skull and scapular parameters can be used to estimate body length with coefficient of determination (*R* ^2^) of 0.985, while scapular morphology using the caudal border tuberosity and coracoid process showed a 91.30% accuracy rate for identifying males and a 96.15% accuracy rate for identifying females. Additionally, we noted that the age range for dugongs can be predicted based on the presence or absence of deciduous tusks. Overall, we have shown that skull morphology and morphometric measurements and scapular morphology can be used as a tool for sex identification, habitat identification and body length estimation.

### Skull/scapula and sex identification

In other species (e.g. human, canine and leopard) the skull can be used for sex identification, either from reference hallmarks or a morphometric equation^8, 28–30^. In this study we showed that sexual dimorphisms can clearly separate males and females by using large permanent tusk morphology, scapular morphology and a skull morphometric equation. Large permanent tusks can be used as a tool for sex identification with a 100% accuracy rate; the scapula could be used to distinguish males and females with 91.3% and 96.1% accuracy, respectively; and skull morphometrics (using parameters from the cranium and mandible) showed a 96.7% accuracy rate. Our study did not find any significantly different parameters of the skull between sexes, but most parameters were higher in females. Scapular width and caudal scapular notch width was greater in females than in males (p* < *0.05), but a morphometric equation from scapular parameters gave a poor accuracy rate (68.1%). A previous study^18^ reported that 6 out of 26 parameters were significantly different between sexes, but only the anterior snout width was larger in males; the other 5 parameters (snout length, pterygoid-frontal depth, anterior mandible depth, extra-mandibular chin width and mandibular length) were significantly higher in females. However, we noted that the previous study had overlapping data, without a clear cut-off value, so this feature may not provide complete separation between sexes. A discrimination function is the best tool for use in sex identification in case large tusks are absent or cutting through tusks is prohibited. We created a discrimination function from three different data sets: cranium, mandible and skull. The results show that skull morphometrics had the highest accuracy (96.7%) and precision (96.9%) rate, while using only parameters from the cranium or mandible did not give higher accuracy and precision. The morphometrics of the dugong skull gave an effective equation for sex identification, as shown in Table 5. Dugong scapulas also exhibited sexual dimorphism that can be used for identifying sex with a high accuracy rate as well.

### Skull/scapula and habitat identification

In many species (e.g. lion, leopard and sea turtle), an animal’s habitat has an effect on skeleton size^11, 28, 31, 32^. Our study also looked for a relationship between skull morphology and location. We found that skulls from dugongs living in the Andaman Sea seemed to be larger than skulls from dugongs living in the Gulf of Thailand. This study found that the base width of the cranium, zygomatic arch length, premaxillae angle of the cranium and mandibular condyle width of dugongs from the Andaman Sea were significantly higher compared with dugongs from the Gulf of Thailand. Only mandibular condyle length was significantly higher in dugongs from the Gulf of Thailand. Also, the minimum width of the scapula in dugongs living in the Gulf of Thailand was significantly lower (p* < *0.05) compared with dugongs living in the Andaman Sea.

After analyzing the discrimination functions from four different data sets – cranium, mandible, skull and scapula – the skull was found to show the highest accuracy and precision rates, of 100%. But the cranium and mandible also showed high accuracy rates of over 90%, while the scapula showed the lowest accuracy rate (73.7%). The morphometrics of the dugong skull provided an effective equation for habitat identification, as shown in Table 7.

### Skull/scapula and body length estimation

Body length in Sirenia is widely used for estimating body weight^33, 34^ and maturation^22, 35^. A previous study demonstrated a correlation between skull measurements, body length and field weight in Florida manatees^36^. However, only four parameters were used, including basal skull length, occipital condyle width, foramen magnum width and foramen magnum height. The highest correlation (R^2^ = 0.90) was between foramen magnum width and field weight. Spain and Heinsohn^25^ studied the correlation between 26 parameters from the skull and body length in 52 dugong samples from Australia (26 mature and 26 immature), and found the best parameter to be condylo-premaxillary length. Their proposed equation was: Y = 54.214 + 1.650X + 0.110X^2^, where Y is the body length from the tip of the snout to the notch in the tail fluke and X is the condylo-premaxillary length, both in centimeters. However, the authors did not report the correlation rate from this equation. In our study, 57 parameters were used (25 from the cranium, 23 from the mandible and 9 from the scapula) and four models were generated from various parameters of the cranium, mandible, skull and scapula. A model from the skull to estimate body length using 12 parameters showed the highest correlation (98.5%) compared with the other three models, as shown in Table 8.

Not only skull morphology can be used as a tool for estimating dugong body length. Burgess *et al*.^35^ reported that male dugongs with body length lower than 240 cm usually had unerupted tusks, while erupting tusks are usually found in dugongs whose body length is approximately 240–259 cm, and dugongs with body length over than 260 cm typically have erupted tusks. However, our study did not find this correlation; but we did find a correlation between tusk length and body length, i.e. there was a low correlation with body length.

### Study Limitations

Our study had many limitations. First, we only had a small number of dugong samples from the Gulf of Thailand, conforming to a previous study which reported few dugongs living in the Gulf of Thailand compared with the Andaman Sea^4, 34, 37^. Only 11 animals from the Gulf of Thailand were used to create an equation to differentiate between the two different locations; thus there was a low power of confidence, even though the result showed very high accuracy (100%). However, it is possible that dugongs living in the two different locations had different skull morphometrics, because the average body length of 69 dugongs taken from the Andaman Sea (2.20 ± 0.48 m) and 11 dugongs taken from the Gulf of Thailand (2.21 ± 0.28 m) was not significantly different. Dugong age was also a limitation; we did not know the true age of these animals, so our study was confined by these two limitation in finding a correlation between skull morphometrics and age. Other studies have tried to estimate dugong maturation based on body length, which defined mature size as longer than 2.2–2.4 m^18, 27^. However, the 44 stranded dugongs used in this study which were found around the coast of Thailand had a lower average size (2.16 ± 0.51 m) compared with those reports^18, 27^. Hence, it is possible that dugongs from different habitats also had a different average size. A previous study reported that out of a total of 101 stranded dugongs in Thailand^34^, 47.5% (48/101) were 2.0–3.0 m long, 31.7% (32/101) were 1.5–2.0 m long and 20.8% (21/101) were less than 1.5 m long. The last limitation was obviously seen when we categorized dugong body length into six groups: lower than 1 m (n = 1), 1.00–1.50 m (n = 2), 1.51–2.00 m (n = 27), 2.01–2.50 m (n = 26), 2.51–3.00 m (n = 23) and over 3 m (n = 1). The distribution of dugong body length was not equal; only one dugong was longer than 3 m and only one was shorter than 1 m (see details in Supplement 1). Nevertheless, we strongly believe that our study of dugong body length showed high reliability because of the large number of dugong skeletons, possibly serving as an accurate representation of a real dugong population.

### Take-Home Message

Study of the morphology of the dugong skull, scapula and other bones can provide useful information in relation to dugong biology, conservation and anatomy. However, the main limitation for studying dugongs is the number of bone samples available in collections and reliable recorded data of these samples. In addition to the presence or absence of deciduous tusk, another one of our ideas that we would like to share with all scientists is to estimate age using skull and scapular morphology and morphometric parameters, because age estimation of stranded dugongs is an important issue for ecology, biology and conservation. In many kinds of animals, studies have used bone morphology or morphometrics for age estimation. Stansfield demonstrated using skull morphology to estimate African elephant age by using an age reference line^12^. In dugongs, research has shown many parameters related to age, such as incisor dentinal growth layer groups^38^, body length^39^ or closure of cranial sutures^40^. However, there is always a question about estimating the real age of dugongs. Our results showed that an equation or model created from many skull parameters could be used for sex and habitat identification and body length estimation, with a very high accuracy rate. We also believe that skull morphology can be used to estimate age and will give a high accuracy rate as well. However, to create this model we had to have dugong skulls for which we knew the actual ages. In contrast, scapular morphometrics showed poor discrimination function but presented excellent hallmarks for sex identification. Studies of other bones might reveal some surprising results and provide valuable insights for dugong biology, conservation and forensic science.

## Conclusion

From this study we learned that dugong skull and scapular morphology and morphometrics can be used for sex and habitat identification as well as body length estimation. A discrimination function from skull morphometrics and two markers on the scapula from our study can be used at both the field and laboratory level for dugong conservation and forensic science. We also noted that a discrimination function that used many parameters from the cranium and mandible gave a better result and accuracy rate than using a single bone or single parameter. Due to the size of bones used in this study and the outcome of all measurement results, we believe that dugongs living in the Gulf of Thailand are of smaller size than dugongs living in the Andaman Sea. But to prove this hypothesis, further studies need to be performed.

## Electronic supplementary material

Supplement 1
