# Supplementary material for: What the skull and scapular morphology of the dugong (Dugong dugon) can tell us: sex, habitat and body length?
Source: Sci Rep. 2017 May 16;7:1964. doi: 10.1038/s41598-017-01899-7 (PMC5434023; doi:10.1038/s41598-017-01899-7)
Supplement: Supplementary file 1 — Supplement 1 [file 41598_2017_1899_MOESM1_ESM.pdf]

What the skull and scapular morphology of the dugong (*Dugong dugon*) can tell us: sex, habitat and body length?

Korakot Nganvongpanit, Kittisak Buddhachat, Patcharaporn Kaewmong, Phaothep Cherdsukjai & Kongkiat Kittiwatanawong

Supplement 1 Samples information

	Sample ID (DUxxx)	Sex		Body length (meter)	Habitat	
		Male	Female		Andaman Sea	Gulf of Thailand
1	014	1		1.70	1	
2	016	1		2.54	1	
3	036		1	2.73	1	
4	037		1	1.70	1	
5	038	1		2.57	1	
6	040	1		2.94	1	
7	043		1	2.14		1
8	047	1		2.21	1	
9	048		1	2.71		1
10	049	unknown		2.26	1	
11	050	unknown		unknown	1	
12	057		1	2.56	1	
13	058	1		2.50	1	
14	059	1		2.45	1	
15	060	1		1.92	1	
16	070	1		2.00	1	
17	074	1		2.06	1	
18	075	1		2.40	1	
19	078		1	2.31		1
20	088	1		2.14		1
21	098	1		2.14		1
22	103	1		1.67	1	
23	119	1		1.53	1	
24	126		1	2.00	1	
25	129		1	2.25	1	
26	130	1		2.09		1
27	143		1	2.00	1	
28	144	1		2.64	1	
29	145	1		1.56	1	
30	146	1		2.14	1	
31	209	unknown		1.60		1
32	227	1		2.47	1	

33	229	unknown		1.80	1	
34	233		1	2.64	1	
35	234		1	2.57	1	
36	236		1	2.54	1	
37	241		1	2.29	1	
38	243		1	2.57	1	
39	248		1	1.91	1	
40	250		1	2.38		1
41	258		1	2.31		1
42	260		1	1.74	1	
43	268	1		1.92	1	
44	270	1		1.60	1	
45	272	1		2.45		1
46	273	unknown		1.80	1	
47	283		1	3.00	1	
48	285		1	2.00		1
49	286	1		1.61	1	
50	287		1	1.91	1	
51	291		1	2.63	1	
52	292		1	2.82	1	
53	293	1		190	1	
54	297		1	1.66	1	
55	299		1	2.81	1	
56	300		1	2.24	1	
57	301	1		2.44	1	
58	306	1		2.02	1	
59	307		1	2.78	1	
60	322	1		2.42	1	
61	323	1		1.92	1	
62	328		1	2.59	1	
63	329		1	1.63	1	
64	330		1	256	1	
65	332	1		1.94	1	
66	336	1		2.00	1	
67	337	1		2.81	1	
68	339	1		1.16	1	
69	341		1	2.67	1	
70	344		1	1.60	1	
71	345	1		2.52	1	
72	346	1		2.14	1	
73	347		1	2.58	1	
74	349		1	2.35	1	
75	350		1	1.38	1	
76	354		1	2.04	1	

77	358		1	1.97	1	
78	359	1		2.11	1	
79	361		1	0.88	1	
80	364	1		3.64	1	
81	366		1	2.55	1	
